# Correction: Effects of psychogenic stress on oxidative stress and antioxidant capacity at different growth stages of rats: Experimental study

**DOI:** 10.1371/journal.pone.0306774

**Published:** 2024-07-05

**Authors:** The PLOS ONE Staff

## Notice of Republication

This article was republished on April 23, 2024, to correct its type. It was erroneously published as a registered report protocol instead of a research article. The publisher apologizes for this error. Please download this article again to view the correct version. Both the originally published, uncorrected article and the republished, corrected article are provided here for reference.

## Supporting information

S1 File Originally published, uncorrected article. (PDF)

S2 File Republished, corrected article. (PDF)
