# Supplementary material for: Effects of psychogenic stress on oxidative stress and antioxidant capacity at different growth stages of rats: Experimental study
Source: PLoS One. 2024 Apr 16;19(4):e0287421. doi: 10.1371/journal.pone.0287421 (PMC11038576; doi:10.1371/journal.pone.0287421)
Supplement: Attachment — Submitted filename: Response to Reviewers.docx [file pone.0287421.s001.docx]

**Review Comments to the Author**

Reviewer #1:

I assume the ethical approval has been obtained prior to this study.

A very informative piece of study, explaining the effects of the relevant factors in the process, therefore making this research applicable to various and different scientific subjects such as biology, medicine, psychology, neurodevelopmental science, aging and even the field of cosmetic science.

I found it relevant and useful in the field of neurodevelopmental sciences and effects on different stages of life.

Looking forward to more helpful studies by these authors as long as the ethical approval is secured.

Reviewer #2:

Effects of psychogenic stress on oxidative stress and antioxidant capacity at different growth stage

This is an important study that addresses the effect of psychogenic stress on the growth of an organism. Results of this study show that effects vary with the growth stage

1. The results presented are original

2. Results presented in pre-print.

3. Experiments are satisfactory done

4. Conclusions are in keeping with the data

5. The sufficiently presented in Standard English and intelligible fashion

6. Ethical approval was obtained

General comments

The title seems incomplete. I suggest the authors to rewrite as

experimental study of the Effects of psychogenic stress on oxidative stress and antioxidant capacity at different growth stages of rats

Under materials and methods, the subtitle “materials is” may be not appropriate for the experimental animals. I suggest that it is removed. The weight of the animals should be included in this section. Therefore the subtitle of body weight may be removed and remain with the subtitle blood collection instead of” blood draw”

Under blood collect include the amount of blood that was collected

In the methods section under the sub title of ““Organ evisceration” the sentence the liver and adrenal were removed seems to be incomplete. Was it placed in a any solution before analysis?

Under methods in the analysis section include the set confidence interval

What is the strength of this study?

Include limitations to this study

What are the recommendations of the study?

**Correction (Title)**

➤ Effects of psychogenic stress on oxidative stress and antioxidant capacity at different growth stages of rats: Experimental study

**Correction and addition (methods)**

➤Methods

➤Experimental animals

**Correction and addition (methods)**

➤Blood collection and anesthesia

➤ The experimental animals were weighed using a digital scale both before and after the experiment. The experimental animals were immobilized using a rat fixation adjuster both before and after the experiment, and 1 ml of blood was collected from the tail vein, respectively. Blood was collected from the tail vein both before and after the experiment and centrifuged at 3,000 rpm for 15 minutes. The centrifuged serum was then transferred into micro tubes and stored in a deep freezer at −80 ℃ until analysis.

The anesthesia was prepared with 25 ml of three types of mixed anesthetic agents (Medetomidine Hydrochloride 1.875 ml+ Midazolam 2 ml + Butorphanol Tartrate 2.5 ml + Normal Saline 18.625 ml) and administered intraperitoneally (0.5 ml / BW100 g) after blood collection at the end of the experiment. To reduce pain, blood was drawn quickly, and anesthesia was checked by skin sensation with a needle.

**Addition (methods)**

➤The removed liver and adrenal glands were immersed in 10% neutral buffered formalin solution and stored until analysis. For analysis, they were washed in saline, trimmed, and wiped dry before wet weight was determined.

**Correction and addition** **(methods)**

➤ The confidence interval was set at 95% and the statistical significance level was set at p < 0.05.

**Correction and addition (Results)**

➤ 95% confidence interval added

**Addition (discussion)**

➤ This study reached its basic findings by investigating both the morphological and biochemical effects of psychogenic stress at different ages on oxidative stress and antioxidant capacity. The PS and age characteristics of the present study’s subjects can be considered to be fundamental data that may make the study adaptable to humans. On the other hand, one limitation of this study is that we were unable to examine the effects of gender characteristics and diet on oxidative stress and antioxidant capacity. Those will be the subject of the next study.

Reviewer #3:

The study by Dr Lee et al describes of changes in reactive oxygen metabolites/species and anti-oxidant potential/capacity in the serum of rats exposed to strong psychogenic stress at different life stages.

The study addresses an important question a show the bodily stressors connect to cellular stress responses.

However, the methods are not precisely described and the measurement of somehow too general.

For instance, the RWIS procedure is not described in detail. As the size of the animals is increasing with the age it is also necessary to give the dimensions of the restrainers used throughout the experiments. It is also not mentioned how the control animals were maintained and were they restrained for the blood withdrawal. The specific details of the anesthetic mixture and the precise dose should also be given. The details of blood treatment should also be described.

There are much more updated, state-of-the-art methods to specify reactive oxygen species which are respond to psychogenic stress.

Finally, changes in body and adrenal indicate the success of the stress exposure, direct measurement of stress hormones (corticosterone, ACTH) and the relation between d-ROM BAP and inflammatory mediators would have been highlighted the significance of the study.

**Addition (methods)**

➤ (CLEA Japan, Inc. japan)

**Addition (methods)**

➤ The RWIS used small animal fixation adjusters (sanplatec.co.japan) with adjustable lengths to account for individual differences in age and body weight (body size). Rats were placed in the “rat fixation adjuster” that adjusts to three sizes of experimental rats (small: Φ31 x 20~80 mm, medium: Φ60 x 60~170 mm, large: Φ76 x 70~220 mm). The intake port at the front of the fixation adjuster was moved to the tail to the maximum setting, and the rats were restrained. The restrained rats were then transferred to a tank with a 2-cm water level and submerged. Since the age of this study’s subjects ranged from youth to old age, fasting was not performed to eliminate the effects of food stress.

**Correction and addition (methods)**

➤ The experimental animals were weighed using a digital scale both before and after the experiment. The experimental animals were immobilized using a rat fixation adjuster both before and after the experiment, and 1 ml of blood was collected from the tail vein, respectively. Blood was collected from the tail vein both before and after the experiment and centrifuged at 3,000 rpm for 15 minutes. The centrifuged serum was then transferred into micro tubes and stored in a deep freezer at −80 ℃ until analysis.

The anesthesia was prepared with 25 ml of three types of mixed anesthetic agents (Medetomidine Hydrochloride 1.875 ml+ Midazolam 2 ml + Butorphanol Tartrate 2.5 ml + Normal Saline 18.625 ml) and administered intraperitoneally (0.5 ml / BW100 g) after blood collection at the end of the experiment. To reduce pain, blood was drawn quickly, and anesthesia was checked by skin sensation with a needle.

**Addition (discussion)**

➤ This study reached its basic findings by investigating both the morphological and biochemical effects of psychogenic stress at different ages on oxidative stress and antioxidant capacity. The PS and age characteristics of the present study’s subjects can be considered to be fundamental data that may make the study adaptable to humans. On the other hand, one limitation of this study is that we were unable to examine the effects of gender characteristics and diet on oxidative stress and antioxidant capacity. Those will be the subject of the next study.

**Reviewer #4:**

The study is an original experimental research. The article was written in standard English, and precise language was used. Experimental results are explained in the manuscript by comparing them with the results of the studies in the literature. Experimental groups (control and others) are sufficient in number. The experimental protocol (RWIS method), as well as the adequacy of the sample size, can be explained in more detail.

The source protocols stated that the subjects were immersed in water after 24 hours of fasting. In this study, were the experimental animals (PS group) fast before being immersed in water? Is 24-hour fasting included in the PS protocol?

If immersed in water after 24 hours of fasting, it would be appropriate to explain the effect of this fasting period on subjects in the growth phase.

**Addition (methods)**

➤Added RWIS method.

➤ The number of animals in each group was set at six to account for mortality during the experiment and stress caused by the rearing environment.

**Addition (methods)**

➤ Since the age of this study’s subjects ranged from youth to old age, fasting was not performed to eliminate the effects of food stress.

Other changes (addition of authors)

➤Yoshihiko Shibukawa ^2^
