# Supplementary material for: Effects of psychogenic stress on oxidative stress and antioxidant capacity at different growth stages of rats: Experimental study
Source: PLoS One. 2024 Apr 16;19(4):e0287421. doi: 10.1371/journal.pone.0287421 (PMC11038576; doi:10.1371/journal.pone.0287421)
Supplement: Attachment — Submitted filename: Response to Reviewers.docx [file pone.0287421.s002.docx]

PONE-D-23-17434R1

Effects of psychogenic stress on oxidative stress and antioxidant capacity at different growth stages of rats: Experimental study

**Additional Editor Comments:**

A more detailed description of the methods, like d-ROM and BAP measurement, should be added.

➤Correction

The assay method was as follows: The d-ROMs assay kit cuvette (4.8PH acetate buffer) is placed in a thermostat space set at 37°C and allowed to warm for 10 minutes. Then, 20 µℓ of thawed plasma is added to the cuvette and mixed by inverting gently up and down for 10 seconds; 20 µℓ of d-ROMs coloring solution Chromogen (N,N diethyl paraphenylenediamine) is used and mixed by gently moving it up and down for 10 seconds in a cuvette. Afterwards, the cuvette is placed in the thermostat space for 5 minutes and measured with a photometer at 505 nm [11,21].

➤Correction

The BAP assay kit cuvette (Thiocyanate HCl) is warmed in the thermostat space set at 37°C for 10 minutes; 50 µℓ of BAP coloring solution (trivalent iron salt) is taken, added to the cuvette, and mixed gently for 10 seconds. The cuvette is placed in the thermostat space for 3 seconds and measured at 505 nm with a photometer; 10µℓ of thawed plasma is placed in the cuvette and mixed gently for 10 seconds. The cuvette is then placed in the thermostat space and incubated for 5 minutes, and again measured with a 505 nm photometer for 3 seconds [11,21].

English editing of the manuscript is highly needed as several typographic, grammatical, and overall style errors still exist.

➤Correction

The composition of the anesthetic should be described as the concentration of the anesthetic agent and then as mg/kg.

➤Correction

The anesthesia was prepared with 25 ml of three types of mixed anesthetic agents (Medetomidine Hydrochloride 7.5µg; 0.375 mg/kg + Midazolam 0.4 mg; 2.0 mg/kg + Butorphanol Tartrate 0.5 mg;2.5 mg/kg) and administered intraperitoneally (0.5 ml / BW100 g) after blood collection at the end of the experiment.

In the conclusion section: ""investigating both the morphological and biochemical effects". Clarify what you meant by morphological effects.

➤Correction

The study reached its basic findings by investigating both the biochemical effects of psychogenic stress on oxidative stress and antioxidant capacity at different ages and the morphological effects on body weight and organs.
